# Investigating the Genetic Basis of Theory of Mind (ToM): The Role of Catechol-*O*-Methyltransferase (COMT) Gene Polymorphisms

**DOI:** 10.1371/journal.pone.0049768

**Published:** 2012-11-27

**Authors:** Haiwei Xia, Nan Wu, Yanjie Su

**Affiliations:** Department of Psychology, Peking University, Beijing, China; Rikagaku Kenkyūsho Brain Science Institute, Japan

## Abstract

The ability to deduce other persons' mental states and emotions which has been termed ‘theory of mind (ToM)’ is highly heritable. First molecular genetic studies focused on some dopamine-related genes, while the genetic basis underlying different components of ToM (affective ToM and cognitive ToM) remain unknown. The current study tested 7 candidate polymorphisms (rs4680, rs4633, rs2020917, rs2239393, rs737865, rs174699 and rs59938883) on the catechol-O-methyltransferase (COMT) gene. We investigated how these polymorphisms relate to different components of ToM. 101 adults participated in our study; all were genetically unrelated, non-clinical and healthy Chinese subjects. Different ToM tasks were applied to detect their theory of mind ability. The results showed that the COMT gene rs2020917 and rs737865 SNPs were associated with cognitive ToM performance, while the COMT gene rs5993883 SNP was related to affective ToM, in which a significant gender-genotype interaction was found (*p* = 0.039). Our results highlighted the contribution of DA-related COMT gene on ToM performance. Moreover, we found out that the different SNP at the same gene relates to the discriminative aspect of ToM. Our research provides some preliminary evidence to the genetic basis of theory of mind which still awaits further studies.

## Introduction

Attributing mental states and emotions of other people is a central ability in our social interaction which has been termed ‘theory of mind (ToM)’ [1]. At the age of about 14–18 months, the human infant begins to understand the mental states of desire, intention, and the causal relation between a person's emotions and goals [2]. Further, a child cannot distinguish between his or her own and others' beliefs and knowledge of the world until the age of 4-year-old [3]. Although there are clear environmental effects on the developmental process of ToM ability, the ontogeny of the ToM faculty follows a distinct sequence of acquisition; these developmental steps of ToM constitute a stereotypical and cross-culturally universal schedule [4]. Naturally, whether there is some genetic basis behind the ToM developmental schedule comes into our considerations.

Twin study showed that ToM performance is highly heritable during the preschool years [5]. The studies of individuals with autism whose ToM ability were impaired also suggest some genetic basis behind the mentalizing process [6]. However, little has been done regarding genetic basis of ToM inheritance. It is widely accepted that ToM can be divided into cognitive and affective aspects, cognitive ToM refers to the ability to make inferences about beliefs and motivations, while affective ToM refers to the ability to infer what a person is feeling [7]. The dissociation between cognitive and affective ToM has been highlighted in clinical studies, for example, in Asperger syndrome, the affective component has been shown to be more impaired than the cognitive one [8]. The same pattern has been observed in schizophrenia, particularly in patients with negative symptoms like abulia and social withdrawal [9]. Neuroanatomical studies suggest that these two aspects of ToM could be mediated by dissociated brain region [10]. For instance, the dorsal striatum, dorsal ACC, dorsal MPFC and the DLPFC are uniquely involved in processing cognitive ToM tasks (e.g., false belief and second-order deception tasks). Constantly, the ventral striatum, the ventral ACC, the ventral MPFC, the OFC and the amygdale are associated with affective ToM (e.g., white lie and faux pas tasks). The available evidence provides us with a constructive model to understand the different dimensions of ToM as well as the neural mechanism of different aspects, and also hints that each aspect of ToM may have a unique genetic basis that need to be explored in greater depth.

**Table 1:** Mean score and standard deviations of every ToM task.

ToM tasks	All(*n* = 101)		Male(*n* = 45)		Female(*n* = 56)	
	Mean	*SD*	Mean	*SD*	Mean	*SD*
Second-order FB	2.13	0.59	2.16	0.61	2.11	0.59
Double bluff	1.57	0.48	1.50	0.52	1.63	0.44
White lie*	2.97	0.93	2.74	0.99	3.15	0.84
Faux pas task	4.42	2.11	4.24	2.02	4.55	2.19
Cognitive ToM	3.70	0.84	3.66	0.86	3.73	0.83
Affective ToM*	7.39	2.46	6.99	2.45	7.71	2.45
Total ToM	11.08	2.75	10.64	2.83	11.44	2.65

Note: *p<0.05.

Turning to the molecular genetics study of ToM, dopamine (DA) is probably the most popular candidate neurotransmitter. For example, functional neuroanatomical changes within dMPFC which is also a major target of mesocortical DA projections have been associated with cognitive ToM development [11]. DA activity promotes plasticity necessary for adjusting expectations and coming to increasingly refined understandings of the causal structure of a given event, and thus mediates mental state reasoning [12]. Brunet-Gouet and Decety also suggested that social cognition may be affected by dopaminergic levels, especially when mentalization is performed [13]. Recent research has shown that individual differences in DA functioning (as measured by eye blink rates) predict preschoolers' theory of mind understanding [14].

**Table 2:** COMT SNPs genotype frequency.

SNP	Genotype	mAF	Frequency	n	Total	*p*-HWE
rs4680	AA/AG/GG	0.470	0.347/0.356/ 0.287	35/36/29	100	0.006
rs4633	CC/CT/TT	0.435	0.495/0.327/ 0.178	50/33/18	101	0.006
rs2020917	TT/CT/CC	0.337	0.505/0.317/ 0.178	51/32/18	101	0.003
rs2239393	AA/AG/GG	0.340	0.574/0.158/ 0.257	58/16/26	100	0.000
rs737865	TT/CT/CC	0.485	0.386/0.257/ 0.356	39/26/36	101	0.000
rs174699	CC/CT/TT	0.195	0.624/0.347/ 0.020	64/35/2	101	0.251
rs5993883	GG/GT/TT	0.386	0.416/0.396/ 0.188	42/40/19	101	0.098

Notes: mAF, minor allelic frequency; *P*-HWE, *P-value* of Hardy–Weinberg equilibrium.

Several genes are known to affect levels of synaptic DA. The enzyme catechol-*O*-methyltransferase (COMT) metabolizes dopamine and is one such influence. COMT gene has been primarily investigated by several researchers. They found that COMT activity was genetically influenced, with the greatest variance explained by a common single nucleotide polymorphism (SNP), Val158Met (rs4680), which refer to the codon 158 of COMT gene, one containing methionine and the other having valine at this position. The 158Val form is a less active enzyme and leads to increased extracellular DA concentrations [15]. There is an approximately 35% enzyme activity difference between the 158Val and the 158Met homozygote in human brain [16].

**Table 3:** ANOVA results for ToM by genotype and gender/genotype interaction.

	Cognitive ToM		Affective ToM		Total ToM	
	GME (*η^2^*)	GGI (*η^2^*)	GME (*η^2^*)	GGI (*η^2^*)	GME (*η^2^*)	GGI (*η^2^*)
rs4680	0.695	1.353	0.108	0.028	0.217	0.195
	*p* = 0.502	*p* = 0.263	*p* = 0.898	*p* = 0.972	*p* = 0.806	*p* = 0.823
	(0.015)	(0.028)	(0.002)	(0.001)	(0.005)	(0.004)
rs4633	0.153	1.670	1.892	2.237	0.949	2.080
	*p* = 0.858	*p* = 0.194	*p* = 0.156	*p* = 0.112	*p* = 0.391	*p* = 0.131
	(0.003)	(0.034)	(0.038)	(0.045)	(0.020)	(0.042)
rs2020917	**3.949**	0.096	0.259	0.511	0.480	0.772
	***p*** ** = 0.023**	*p* = 0.908	*p* = 0.772	*P* = 0.602	*p* = 0.620	*p* = 0.465
	**(0.077)**	(0.002)	(0.005)	(0.011)	(0.010)	(0.016)
rs2239393	1.082	0.063	1.024	1.236	1.149	1.170
	*P* = 0.343	*P* = 0.939	*p* = 0.363	*p* = 0.295	*p* = 0.321	*p* = 0.315
	(0.023)	(0.001)	(0.021)	(0.026)	(0.024)	(0.024)
rs737865	**3.902**	1.192	1.447	0.156	1.712	0.068
	***P*** ** = 0.024**	*p* = 0.308	*p* = 0.241	*p* = 0.856	*p* = 0.186	*p* = 0.934
	**(0.076)**	(0.024)	(0.030)	(0.003)	(0.035)	(0.001)
rs174699	0.761	1.657	0.677	1.114	1.260	2.291
	*p* = 0.470	*p* = 0.201	*p* = 0.511	*p* = 0.294	*p* = 0.288	*P* = 0.133
	(0.016)	(0.017)	(0.014)	(0.012)	(0.026)	(0.024)
rs5993883	1.454	1.762	2.619	**3.346**	**3.218**	2.279
	*P* = 0.239	*P* = 0.177	*p* = 0.078	***p*** ** = 0.039**	***p*** ** = 0.044**	*p* = 0.108
	(0.030)	(0.036)	(0.052)	**(0.066)**	**(0.063)**	(0.046)

Notes: GME, genotype main effect; GGI, gender/genotype interaction.

Bassett and colleagues examined the role of COMT gene functional Val158Met (rs4680) allele in schizophrenia-related expression in 22q11 deletion syndrome (22q11DS); they found that the Met allele hemizygosity showed significantly worse performance in theory of mind task than Val allele group [17]. Thus, an interesting association between COMT gene rs4680 SNP and theory of mind may have emerged. However, negative evidence for this relation has been reported by a recent study. Preschool children were tested, and they used the representational theory of mind (RTM, including false belief contents and false belief location) tasks [18]. They found the COMT rs4680 were not associated with children's RTM performance.

**Figure 1:**
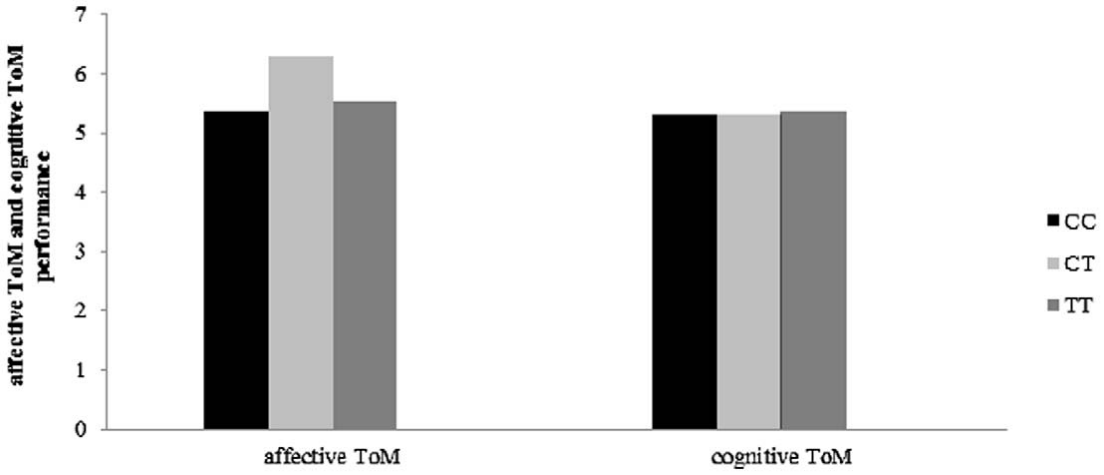
indicated that CT carriers of COMT rs2020917 perform better on cognitive ToM than those with CC genotype (*p* = 0.004) and TT genotype (*p* = 0.043).

The inconsistent findings mentioned above are attributed to several reasons. First, Bassett's experiment involved adults with physiological disease, but Lackner tested healthy preschool children [17], [18]. A recent review indicated that genetic effects become more and more important with development [19]. Thus the age difference between participants of the two studies mentioned above might be helpful in explaining the inconsistent findings to some degree. Second, the experimental paradigm of ToM is different and neither of the studies discriminates the cognitive ToM or affective ToM. Thirdly, it is difficult to imagine the complexity of the tremendous pathway that from single gene polymorphism to human social behavior, they only selected the COMT gene rs4680 SNP, but the DA-related COMT gene contains a lot of functional SNPs, many of them (rs2239393, rs59938883, rs737865, rs4633, rs2020917) on the COMT gene are believed to be the risky genetic elements of schizophrenia [20]–[23], and rs174699 is correlated with drug-use behavior [24]. Another association study pointed out the relations between several COMT SNPs (rs737865, rs5993883, rs4680 and rs4633) and both personality traits and suicidal behavior [25]. Given the importance of theory of mind for healthy social functioning, it is not surprising that individuals with sociability disorders, such as schizophrenia, drug and suicide executants [26]–[28], perform poorly on theory of mind tasks, we assume the seven SNPs on the DA-related COMT gene discussed above (rs4680, rs4633, rs2020917, rs2239393, rs737865, rs174699 and rs5993883) might be associated with ToM ability. Therefore, we designed our study to address the possible specificity of the relations between these COMT gene SNPs and ToM so as to reveal a distinct and stable prospect including COMT gene and cognitive/affective ToM. Since genetic influence in social cognition appears stronger as children grow up [29], and cognitive ToM and affective ToM are mediated by disassociated neural circuit [10], we predict that the relation between COMT gene SNPs and ToM may also show different profile with different aspects of ToM.

**Figure 2:**
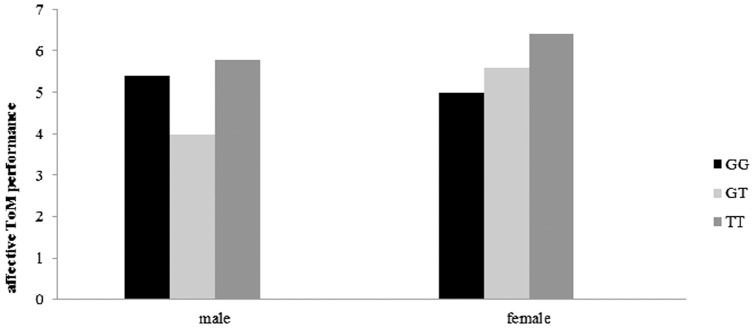
Revealed that female carriers of COMT rs599883 GT genotype scored higher than males with GT genotype (*p* = 0.023). And for females, TT genotype carriers performed better on affective ToM tasks than GG carriers (*p* = 0.017).

On the basis of prior studies, we propose that these functional polymorphisms in COMT gene which promote affect DA levels might be associated with cognitive and affective ToM performance. We invited healthy adults into our research, and we used cognitive and affective ToM tasks to test the ToM ability in order to compare our results with other researchers.

## Methods

### Participants

Participants were all healthy and with no family history of mental problem which included 101 adults (55 females). Age range was from 19 to 27 (*M = *22.50y, *SD* = 2.28). The present experiment was approved by the Ethics Committee of the Psychology department at Peking University, and written consent was obtained from all participants prior to the experiment.

### Measures and materials

The participants first completed 8 short passages with questions assessing their cognitive and affective ToM abilities, and then provided saliva samples for genetic analyses.

### Theory of mind battery

We selected four ToM tasks that could be ordered in terms of developmental complexity and difficulty: second–order false belief, double bluff, white lie and Faux pas, each test consisted of two short stories/passages. The second-order false belief and double bluff tasks tested the cognitive aspect of ToM (cognitive ToM), while the white lie and faux pas tasks assessed the affective aspect of ToM (affective ToM) [30].

#### Second-order false belief tasks [31]

The tasks consisted of two stories, in which participants were asked to predict where one character would think another character would go and why the first character held a false belief about the other character's belief. The total score of the tasks were 0–4 with each story scored 0–2.

#### Double bluff tasks [32]

Double bluff tasks contained a prisoner story and a fan story, in both stories, one of the characters was a big liar, and the other knew this, the liar intentionally told the other character the wrong direction. After the story, two intentional question would be asked “why will X look there for his fan (or the troop)” and “to whom and (where) will X look for his fan (or the troop)”. The total score of double bluff was 0–4 with each task scored 0–2.

#### White lie tasks [33]

The white lie test consisted of two short passages. The first passage was that the character's beloved aunt visited him/her with a new hat, but the character thought the hat was very ugly indeed. When aunt asked the character, “how do you like my new hat”, the character said, “Oh, very nice”. The first question was “was it true what the character said” and the second was “why did he say it”. The situation of the second passage was similar with the first one. The total score of white lie was 0–4 with each passage scored 0–2.

#### Faux pas tasks [34]

The faux pas tasks consisted of 2 short passages each involving a social situation. In the situation, one of the characters said something inappropriate to the situation (a faux pas). Participants must indicate whether somebody said something that they should not have said. If a faux pas was detected, then comprehension of the situation was assessed by asking three further clarifying questions about the faux pas. The first of these clarifying questions concerned who made the faux pas. The second concerned why the protagonist should not have said what she/he said and the third concerned why he/she had said that. Each passage scored 0–6, total score of Faux pas tasks were 0–12.

### Genotyping

DNA was extracted from saliva collected into Oragene saliva kits (DNAGenotek Inc., Beijing, China) using the Agencourt DNAdvance Kit (TianGen Biotech Ltd., Beijing, China). Based on the previous reports, we selected seven SNPs (rs4680, rs4633, rs2020917, rs2239393, rs737865, rs174699 and rs59938883) in the COMT gene through the SNP database (http://www.ncbi.nlm.nih.gov/SNP/). These SNPs were genotyped by polymerase chain reaction–restriction fragment length polymorphism (PCR-RFLP) analysis, and only those with frequencies of minor alleles 5% were used as genetic markers in this study. The MassArray PCR primer and probe sets for the COMT were available by Assays-On-DemandTM (www.sequence.com). They were genotyped using the MassArray genotyping platform (following the manufacturer's protocol) in 5 μl system containing 2.5 μl of GeneAmp® PCR Master mix, 0.25 μl of 20× MassArray®probe, and 1 μl genomic DNA byHotstar Taqr®500. Allele calling was analyzed by Typer4.0 software.

## Results

Table 1 listed the mean score of every ToM task. As anticipated, based on prior research, confirmatory factor analysis (CFA) showed a robust two-factor structure for adults' ToM tasks, and that explains the 60.14% of total variance. The scores of second-order FB and double bluff were added together as the performance of cognitive ToM, and then affective ToM was a combination of white lie and faux pas tasks. There was a significant gender difference on white lie task (*p*<0.05), which may reflect that females performed better in understanding the emotional state of others.

The summary information for the 7 SNPs on COMT gene was reported in table 2, including the minor allele frequencies and *p*-values for the tests of Hardy-Weinberg equilibrium (HWE), which were conducted using Stata's genhwi program [35].

Our focal interest was whether allelic variations in COMT gene would predict the different components of ToM. The ANOVA results for ToM by genotype and gender/genotype interaction was showed on table 3. We found out that four SNPs (rs4680, rs4633, rs2239393, rs174699) in COMT gene were not associated with adults' affective/cognitive ToM performance. However, there were three COMT gene SNPs (rs2020917, rs737865, rs5993883) displayed impact on different components of ToM.

Concretely, there was an association between COMT gene rs2020917 SNP and cognitive ToM performance, *F* (2, 95)  = 3.95, *p* = 0.023, *η^2^* = 0.077. Post-hoc test indicated that adults with CT genotype perform better on cognitive ToM than those with CC genotype (*p* = 0.004) and TT genotype (*p* = 0.043) (see Figure 1). The genotype of COMT gene rs737865SNP was also associated with cognitive ToM performance, *F* (2, 95)  = 3.90, *p* = 0.024, *η^2^* = 0.076. Post-hoc test indicated that adults with CC genotype perform better on cognitive ToM than those with TT genotype (*p* = 0.010). To be worth mentioning, the genotype of COMT rs2020917 and rs737865 didn't contribute to affective ToM performance.

Association between adults' affective ToM performance and COMT gene genotype was found on the COMT rs5993883, which revealed a significant gender-genotype interaction (see Figure 2), *F* (2, 95)  = 3.35, *p* = 0.039, *η^2^* = 0.066. Simple effect test showed females with GT genotype scored higher than males with GT (*p* = 0.023). And for females, TT genotype carriers performed better on affective ToM tasks than GG carriers (*p* = 0.017).

## Discussion

Our study is the first to investigate the genetic basis of adults' affective and cognitive ToM performance. Seven SNPs on the DA-related COMT gene were tested. We found evidence that the COMT rs2020917 and rs737865 were associated with adults' cognitive ToM performance. Moreover, an association between the COMT rs5993883 and affective ToM performance was found. But for the other four COMT SNPs (rs4680, rs4633, rs2239393, rs174699), none of them were associated with affective/cognitive ToM performance. These findings raise the possibility that the DA-related COMT gene plays some role in the process of mental understanding.

For the most popular SNP on the COMT gene, Val158Met (rs4680), evidently, adults' genotypes were not associated with ToM performance in our study. This result was consistent with the previous finding on typically developed children [18]. Ebstein indicated that independent replication is a crucial step in validating both GWA studies and candidate gene investigations [19]. Our Mongoloid participants ethnically diverged from the sample that was largely Caucasian who joined Lackner's research. Despite the presence of largely racial and age difference, we found the same result on the COMT rs4680, this independent and repeated verification indicated that there might be no association between COMT SNP rs4680 and ToM performance.

Previous study had established that general levels of DA are associated with RTM reasoning in preschoolers [14]. In order to recover the precise neurobiological mechanisms for this association, they tested three DA-related genes and found out that common polymorphisms in DRD4, but not COMT or DAT1, were associated with preschool children's RTM performance. But, as we know, COMT metabolizes dopamine and COMT gene products could affect levels of synaptic DA. Therefore, there may be a reasonable association between COMT gene and RTM performance. However, Lackner concentrated on only one SNP (rs4680) in COMT gene. In our study, we tested seven SNPs in COMT gene and found out some significant associations.

It is noteworthy that COMT SNPs modulated different aspects of ToM performance. We found the COMT rs2020917 and rs737865 show significant associations with cognitive ToM performance and the COMT rs5993883 shows a significant association with affective ToM performance. This is an enlightening finding that different SNP at the same gene relate to the discriminative aspect of ToM. Recently, studies indicated that three COMT SNPs (rs2020917, rs737865 and rs5993883) may be the risky genetic factors of schizophrenia. Another association study pointed out to the relationship between COMT SNPs (rs737865, rs5993883) and both personality traits and suicidal behavior [25]. Moreover, studies revealed that individuals with schizophrenia usually have an impaired ToM ability [20]–[23]. In those studies, researchers used first-order and second-order false belief task which was believed to be the cognitive aspect of ToM as the specific measures of ToM ability. Thus, the present study found further genetic mechanisms of ToM deficit by revealing that COMT SNPs (rs2020917, rs737865) might contribute to cognitive ToM. While for the COMT SNP rs5993883, there was evidence implicating a linkage between this SNP and suicidal behavior [25]. To our knowledge, patients with affective disorders have a higher risk of being suicidal. These may emerge as the beginning of some exciting evidence chain, we hope, future studies will help in solving the mystery.

For the functional polymorphism rs4680, we found no association between COMT genotype and gender, which was in line with the findings of Barnes and his colleagues, such that there was no association of COMT (rs4680) genotype with brain structure differences between men and women [36]. The COMT rs4680 polymorphism is not always a sensitive candidate genotype. Besides, for the COMT rs5993883, females with TT genotype performed better on affective ToM tasks than males with TT. From an evolutionary view, women would face a higher cost if cheated by male due to their higher parental investment in potential offspring [37]. One study has found individual differences between male and female preadolescents, where young females outperformed preadolescent boys in their theory of mind abilities and social skills [38]. Our results provide another evidence of gender difference in ToM, especially in affective ToM.

One limitation in our study was the absence of measuring executive function, it should be noted that some social cognitive abilities, such as inhibitory control and general intelligence, might involve into the relations between COMT gene and theory of mind. Besides, the sample sizes from the current study were not so optimal that these results should have to be interpreted with caution when amplified into a larger population. Also, sample restriction might be reflected in the small effect size of COMT gene SNPs in our study. Alternatively, we may not rule out the possibility of systematic errors of genotyping, which caused the disequilibrium and thus probably leading to inflation of Type I error. Other candidate genes might also be considered, as well as gene×gene interactions and gene×environmental interactions. In spite of those limitations, this is the first study to our knowledge that explore the relation between COMT SNPs and the two aspects of ToM. And interestingly, we demonstrate specific DA-relate gene (COMT) was involved in different components of ToM. Future studies should consider more details and draw a whole picture of the genetics basis of mindreading.

## Conclusion

In sum, our goal was to investigate the possibility that the heritable basis of affective and cognitive theory of mind might be in part attributable to COMT gene that affects dopamine level. We found out that the different SNP at the same gene relates to the discriminative aspect of ToM. Our research provided some preliminary evidence and a basis for further research in the area.
